# Absence of Thioredoxin Domain Containing 5 Improves Glucose Tolerance and Insulin Sensitivity in Male Mice

**DOI:** 10.3390/ijms27146286

**Published:** 2026-07-15

**Authors:** Javier Sánchez-Marco, Cristina Barranquero, Roberto Martínez-Beamonte, Joaquín C. Surra, Seyed Hesamoddin Bidooki, Luis V. Herrera-Marcos, María-Jesús Rodríguez-Yoldi, María A. Navarro, Marta Lopez-Yus, Jose M. Arbonés-Mainar, Jesús Osada

**Affiliations:** 1 Departamento de Bioquímica y Biología Molecular y Celular, Facultad de Veterinaria, Instituto de Investigación Sanitaria de Aragón-Universidad de Zaragoza, E-50013 Zaragoza, Spain; javiersanchezmarc@gmail.com (J.S.-M.); h.bidooki94@gmail.com (S.H.B.); angelesn@unizar.es (M.A.N.); 2 Instituto Agroalimentario de Aragón, CITA-Universidad de Zaragoza, E-50013 Zaragoza, Spain; cbarranquero.iacs@aragon.es (C.B.); romartin@unizar.es (R.M.-B.); jsurra@unizar.es (J.C.S.); lherrera@unizar.es (L.V.H.-M.); mjrodyol@unizar.es (M.-J.R.-Y.); 3 CIBER de Fisiopatología de la Obesidad y Nutrición, Instituto de Salud Carlos III, E-28029 Madrid, Spain; jmarbones.iacs@aragon.es; 4 Departamento de Farmacología, Fisiología, Medicina Legal y Forense, Instituto de Investigación Sanitaria de Aragón-Universidad de Zaragoza, E-50013 Zaragoza, Spain; 5 Departamento de Producción Animal y Ciencia de los Alimentos, Escuela Politécnica de Huesca, Instituto de Investigación Sanitaria de Aragón-Universidad de Zaragoza, E-22071 Huesca, Spain; 6 Instituto de Investigación Sanitaria de Aragón, E-50009 Zaragoza, Spain; martalyus@gmail.com

**Keywords:** thioredoxin domain containing 5, TXNDC5, glucose, glycemia, insulin, NEFA, insulin resistance, liver, *Txndc5*-deficient mice, *G6pc*, *Irs2*, *Igfbp1*

## Abstract

Thioredoxin domain-containing protein 5 (TXNDC5) plays a role in diseases related to oxidative stress, energy metabolism, and cellular inflammation. This protein has also been associated with diabetes and insulin folding. To gain insight into these relationships, glucose metabolism was characterized using *Txndc5*-deficient mice. The absence of TXNDC5 lowered glycemia, which was correlated with higher non-esterified fatty acid (NEFA) levels following an overnight fast on a chow diet in males. Several tolerance tests (pyruvate, glucose, and insulin) revealed no impairment in gluconeogenesis, but rather, higher insulin sensitivity. In vitro assays using an engineered hepatic cell line corroborated the results of increased glucose uptake. When a high-fat, high-sucrose diet was administered to induce a prediabetic state, the absence of TXNDC5 reproduced the lower glycemia and higher NEFA levels observed in mice consuming the chow diet. However, higher levels of plasma insulin were observed in *Txndc5*-deficient mice. The insulin receptor was increased in the hepatic plasma membranes. Increased hepatic gene expression of *G6pc*, *Irs2*, and *Igfbp1* was also observed in the absence of TXNDC5. These results indicate that TXNDC5 plays a role in the hepatic sex-differential handling of glucose and lipids, and in retaining the insulin receptor on the plasma membrane.

## 1. Introduction

The protein disulfide isomerase (PDI) family A, member 15 (PDI15), also known as resident endoplasmic reticulum resident protein 46 (Erp46) or thioredoxin domain containing protein 5 (TXNDC5), has three thioredoxin (TRX)-like domains that catalyze its chaperone activity in the endoplasmic reticulum (ER), where it is primarily located [1]. As a PDI, it has been proposed to ensure the proper folding of secreted and membrane proteins, as well as to maintain redox homeostasis in cells and ERs [2].

Several cancer types show TXNDC5 overexpression, potentially driven by tumor hypoxia, which promotes anaerobic metabolism and proliferation and can induce TXNDC5 expression [1,3]. The same mechanism has been proposed in other pathologies such as diabetes [4], arthritis [5,6], neurodegenerative diseases [7], and vitiligo [8]. TXNDC5 has been linked to inflammation in various ways [9,10]. It has also been associated with the extent of fatty liver in *Apoe*-deficient mice fed squalene [11]. Furthermore, the increase in TXNDC5 via glucagon-like peptide-1 treatment could protect against metabolic dysfunction-associated steatotic liver disease by alleviating ER stress [12] since it modulates ER stress signaling pathways [13]. The absence of this protein has been translated into hepatic steatosis and a fasting metabolic stress resembling an acute phase response [14].

In pancreatic β-cells, TXNDC5 has been found to protect against ER stress and apoptosis [4]. It is also one of the five protein disulfide isomerases (PDIs) activated via the IRE1α-XBP1 pathway of the unfolded protein response, which catalyzes the oxidative folding of proinsulin [15]. An interaction between the latter and TXNDC5 was observed using immunoprecipitation and ligation proximity assays [16]. Furthermore, reduced levels of TXNDC5 have been observed in the pancreas of diabetic mice [16]. Using exon sequencing, a correlation between heterozygous TXNDC5 loss of function and an increased risk of type 1 diabetes was reported in humans [17]. Rheumatoid arthritis synovial fibroblast-like cells lacking TXNDC5 showed increased insulin-like growth factor binding protein 1 (IGFBP1) [5] implying that TXNDC5 potentially controls insulin in other tissues. A proteomic approach revealed a sevenfold down-regulation of TXNDC5 in the livers of fructose-fed hamsters, as a model of insulin-resistance [18]. These findings evidence that a direct role of TXNDC5 on hepatic glucose homeostasis is missing and the working hypothesis would be that TXNDC5 deficiency improves or impairs hepatic insulin action through altered receptor processing. To test this hypothesis, the glucose metabolism of *Txndc5*-deficient mice was characterized in the context of the previously reported hepatic steatosis and genetic differences in response to fasting [14].

## 2. Results

### 2.1. Somatometric and Plasma Parameters of Txndc5-Deficient Mice

**Figure 1:**
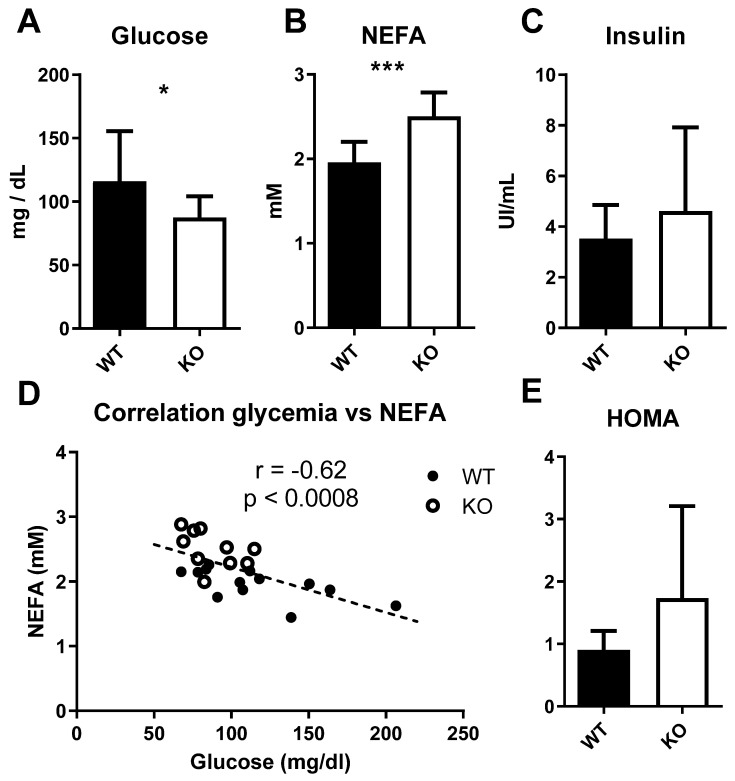
Parameters related to glucose metabolism in males. Plasma glucose (**A**), NEFA (**B**) and insulin (**C**) concentrations. Relationship between plasma glucose and NEFA concentrations (**D**). Homeostatic model assessment, (HOMA) (**E**). Data are means ± SD for each group (n = 13 and n = 10 for WT and KO, respectively). Statistical analyses were done according to Mann–Whitney U-test and *, *p* value < 0.05; and ***, *p* value < 0.001 vs. WT. Spearman’s correlation coefficient and its significance were obtained.

Two groups of two-month-old male and female wild-type (WT) and *Txndc5*-knock-out (KO) mice, obtained and genotyped as previously published [14], were fed a chow diet for 4 weeks. No difference in body weight was observed in the absence of TXNDC5 (Table 1). However, male *Txndc5*-deficient mice needed more energy to gain weight. They also showed greater liver mass and higher hepatic triglyceride and cholesterol levels, reflecting hepatic storage of excess calories. Following a 6 h fasting, no significant changes in plasma cholesterol, glucose, and triglycerides were observed in either sex by the influence of TXNDC5 absence although there were significant differences between both sexes (Supplementary Table S2). However, using a prolonged fasting of 16 h, a significantly lower glycemia was observed in males lacking TXNDC5 (Figure 1A) accompanied by significantly increased NEFA concentrations (Figure 1B), despite the absence of changes in plasma insulin levels (Figure 1C). Glycemia and NEFA levels showed an inverse correlation (Figure 1D). Homeostatic model assessment (HOMA) was not significantly augmented (Figure 1E). There were significant changes between sexes regarding plasma cholesterol, glucose, NEFA and triglycerides (Supplementary Table S3).

### 2.2. Tolerance Tests in Txndc5-Deficient Mice

**Figure 2:**
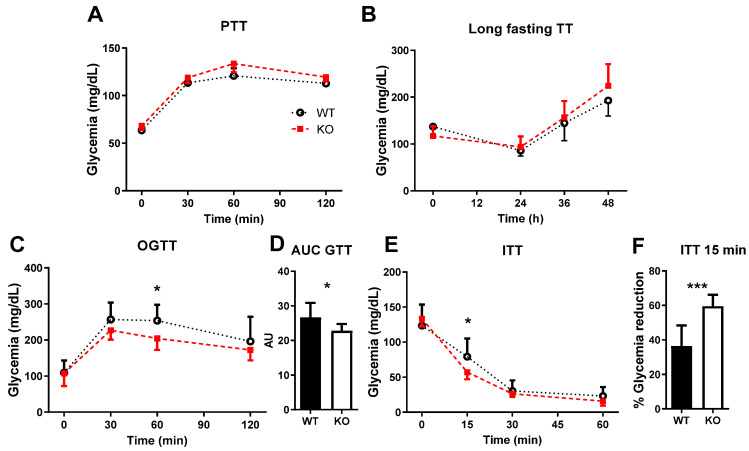
Metabolic challenges on male *Txndc5*-deficient mice. Pyruvate tolerance test (**A**), glucose follow-up during prolonged fasting (**B**), oral glucose tolerance (**C**) and insulin tolerance (**E**) tests in male *Txndc5*-deficient mice (KO) and wild-type (n = 6). The area under the curve of OGTT (**D**) was calculated with no curve fitting, taking baseline as 0. Percentages of glycemia reduction at 15 min taken from ITT data (**F**). Data are means ± SD for each group. Statistical analyses were done according to Mann–Whitney’s U-test to compare specific time points and *, *p* value < 0.05; ***, *p* value < 0.001 vs. WT.

Because insulin levels and HOMA did not change, we next tested whether TXNDC5 affected gluconeogenesis, which can increase after 16 h of fasting [19,20]. A pyruvate tolerance test (PTT) showed no evidence of impaired gluconeogenesis from pyruvate in males lacking TXNDC5 (Figure 2A). Follow-up of plasma glucose over long periods of fasting was conducted to determine whether impaired gluconeogenesis occurred under harsh conditions (Figure 2B). No significant difference was observed in males, in agreement with the results of PTT. Both experiments suggest that gluconeogenesis is not influenced by the absence of TXNDC5.

An oral glucose tolerance test (OGTT) was performed to gain more insight on glucose homeostasis. Males lacking TXNDC5 showed a significant reduction in the 60 min glucose peak and a smaller area under the curve (AUC), confirming that the absence of TXNDC5 promotes glucose utilization (Figure 2C,D). This change was also significant (*p* < 0.025) using two-way repeated measures ANOVA. An intraperitoneal insulin tolerance test (ITT) (Figure 2E) revealed a significant decrease in glucose 15 min after injection in TXNDC5-KO mice when Mann–Whitney’s U-test was used for comparison. This change was not significant using two-way repeated measures ANOVA. When percentage of glucose reduction was calculated (Figure 2F), a significant increase was observed.

Female mice showed no significant TXNDC5-dependent changes after prolonged fasting (Supplementary Figure S1A), nor in the OGTT or its AUC (Supplementary Figure S1B,C). However, the ITT revealed a significant reduction in glycemia 30 min after insulin administration, resembling the response observed in males but at a later time point.

### 2.3. Insulin Sensitivity Following a High-Fat, High-Sucrose Diet (HFHSD) in Txndc5-Deficient Mice

**Figure 3:**
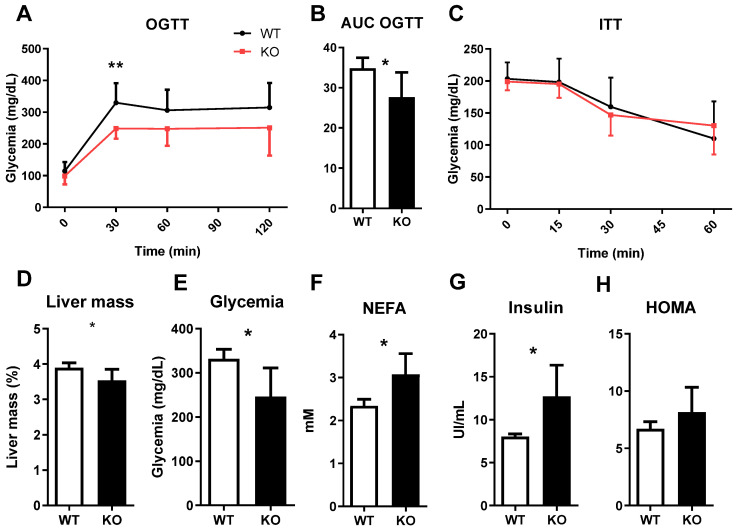
Characterization of *Txndc5*-deficient mice on a high-fat, high-sucrose diet. Oral glucose tolerance test (**A**), area under the curve of OGTT (**B**), insulin tolerance test (**C**), liver mass (**D**), plasma glucose (**E**), NEFA (**F**) and insulin (**G**) were measured after 16 h fasting prior to sacrifice. Calculated HOMA (**H**). Data are means ± SD for each group (n = 6 and n = 7 for WT and KO, respectively). Statistical analyses were done according to Mann–Whitney’s U-test to compare specific time points and *, *p* value < 0.05; **, *p* value < 0.01 vs. WT.

Metabolic disturbances associated with obesity include insulin resistance and hyperinsulinemia, a phenotype that has been reproduced in mice using a HFHSD [21]. To determine the extent to which this phenotype was influenced by the absence of this protein, *Txndc5*-deficient mice were fed a high-fat, high-sucrose (HFHSD) diet. The loss of TXNDC5 did not influence body weight in either sex (Supplementary Figure S2). After two weeks on this diet, an OGTT was performed and the *Txndc5*-deficient mice responded better than the wild-type mice. They exhibited a consistent reduction 30 min after oral glucose administration and a significantly lower area under the curve (Figure 3A,B). However, after four weeks on the HFHSD, an insulin tolerance test showed no differences between groups, as expected due to the insulin resistance caused by this diet (Figure 3C). In females, no difference was observed in the OGTT (see Supplementary Figure S3A), which is consistent with the results from the chow diet. ITT performed in mice lacking TXNDC5 showed no significant difference (see Supplementary Figure S3B). These results highlight the influence of sex on insulin resistance.

Unexpectedly, WT male mice had a higher liver mass (Figure 3D) in contrast with the chow diet. After 16 h fasting, compared to the chow diet (Figure 1A), the HFHSD induced fasting hyperglycemia that was significantly lower in TXNDC5-KO mice (Figure 3E). The latter also showed a significant increase in NEFA (Figure 3F). Plasma insulin significantly increased in TXNDC5-KO mice (Figure 3G), which explains the lower levels of glycemia but contradicts results described in pancreatic β-cell lines [12,16]. HOMA was not significantly increased in the absence of TXNDC5 (Figure 3H). No changes were observed in liver mass and glycemia in females, nor in plasma triglycerides or cholesterol in both sexes (Supplementary Table S4).

### 2.4. Expression of Proteins and Genes Involved in Glucose Metabolism in the Liver

**Figure 4:**
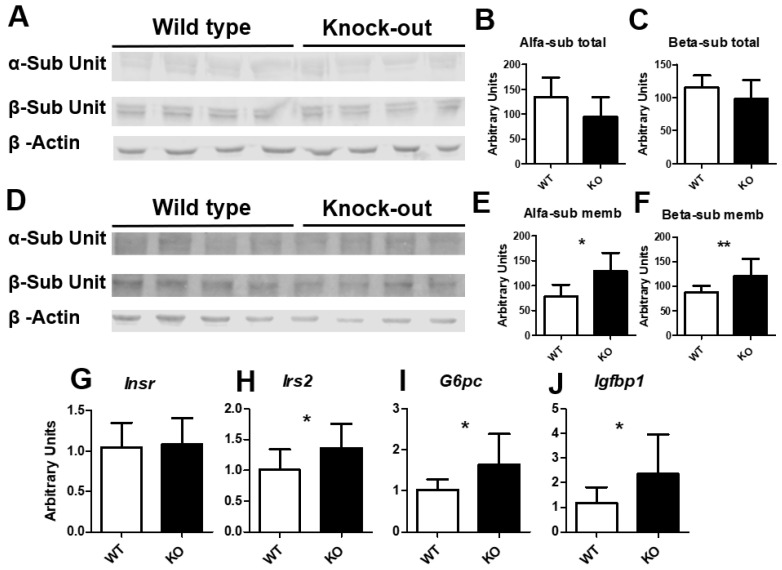
Insulin receptor and associated genes in male mice receiving a chow diet. Representative Western blots of insulin receptor subunits in hepatic homogenate (**A**). Quantification of intensity bands for the alpha subunit (**B**) at 135 kDa and the beta subunit (**C**) at 90 kDa. Representative Western blots of hepatic plasma membrane insulin receptor subunits (**D**). Quantification of intensity bands for the alpha subunit (**E**) at 135 kDa and the beta subunit (**F**) at 90 kDa (n = 4). Hepatic mRNA expressions of *Insr* (**G**), *Irs2* (**H**), *G6pc* (**I**) and *Igfbp1* (**J**) (n = 10 and n = 13 for WT and KO, respectively). Data are means ± SD for each group. Statistical analyses were done according to Mann–Whitney’s U-test and *, *p* value < 0.05; **, *p* value < 0.01 vs. WT.

Glucose homeostasis is controlled by the liver since it stores excess glucose from blood in the form of glycogen and releases glucose through the regulation of glycogenolysis or gluconeogenesis during fasting [22]. As shown above, the absence of TXNDC5 clearly modifies fasting glycemia and oral glucose tolerance tests under chow or HFHSD in males, which requires further molecular insight. When protein levels of both insulin receptor (INSR) subunits were determined by Western blot in the male livers, no significant changes were observed using total liver homogenates (Figure 4A–C). However, higher amounts of both subunits in TXNDC5-KO were observed in the plasma membrane-enriched fractions (Figure 4D–F).

Hepatic gene expressions involved in glucose metabolism were searched in the RNAseq dataset (GSE185515) [14]. Three genes involved in glucose metabolism were found to have differential expressions and confirmed by RT-qPCR: *Irs2*, *Igfbp1* and *G6pc* along with *Insr*. All of these genes, except *Insr*, significantly increased their transcript levels in the livers of *Txndc5*-deficient mice (Figure 4G–J).

### 2.5. Insulin Sensitivity in the Hepatic Cell Line AML12

**Figure 5:**
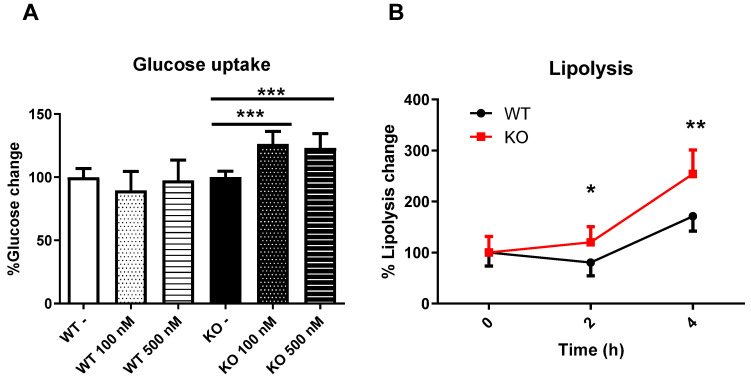
Influence of TXNDC5 on the sensitivity to insulin and isoproterenol in cell culture. (**A**) Glucose uptake was assayed by adding 2-deoxy-d-[3H] glucose to cell media and the cells incubated in the presence or absence of insulin (100 and 500 nM) for 1 h. (**B**) Lipolysis assay was assayed by the release from 9,10-[3H] palmitate in cellular supernatant after 2 and 4 h incubation with isoproterenol. Counts were normalized to protein content in panel (**A**) or TG in panel (**B**). Data are means ± SD for each group (n = 6). Statistical analyses were done according to Mann–Whitney U-test to compare specific time points and *, *p* value < 0.05; **, *p* value < 0.01 vs. WT and ***, *p* value < 0.001 vs. WT.

A murine hepatic TXNDC5-KO AML12 cell line generated using CRISPR-Cas9 technology [23] was used to investigate the impact of TXNDC5 deficiency on insulin sensitivity in vitro. Consistent with previous reports [24,25,26], *Txndc5*-deficient AML12 cells exhibited slower growth under low glucose and low insulin levels. Notably, insulin had no effect on this aspect (Supplementary Figure S4A–C). Furthermore, the lack of TXNDC5 was associated with reduced oxidative stress resistance (Supplementary Figure S4D). When glucose uptake following insulin stimulation was assayed, the *Txndc5*-deficient cell line was sensitive to insulin, showing an increase in glucose uptake (Figure 5A). Unlike muscle and adipose GLUT4, the hepatic GLUT2 transporter is not translocated into the membrane upon insulin stimulation. It is constitutively expressed, and glucose is taken up into the hepatocyte following its phosphorylation [27]. However, insulin shifts the glucose phosphorylation-to-dephosphorylation ratio [28]. Thus, the increased hepatic glucose uptake in KO cells supports previous findings. Isoproterenol activates lipolysis [29,30]. Cells were treated with isoproterenol after incubation with radioactively labeled palmitic acid to investigate this process. Higher lipolysis was observed in the absence of TXNDC5 (Figure 5B), suggesting higher sensitivity to this agent.

## 3. Discussion

The present study aimed to characterize glucose metabolism and insulin sensitivity in mice lacking TXNDC5 and to identify the cellular and molecular mechanisms involved. Our results indicate that only TXNDC5-KO male mice exhibit lower glycemia, which correlates with higher plasma NEFA levels on a chow diet. These mice were subjected to several metabolic challenges on this diet. Despite the absence of changes in pyruvate and prolonged fasting tolerance tests, lower glycemia was observed in KO males subjected to glucose tolerance tests. To investigate how glucose influences insulin metabolism, we administered a prediabetic high-fat, high-sucrose diet. Once again, glucose levels were lower in KO males after a 16 h fast or after glucose tolerance tests. In light of these findings, hepatic insulin receptor analyses showed increased INSR levels in the plasma membranes of TXNDC5-KO males despite no changes in total protein or RNA expression. The expressions of the *Irs2*, *G6pc*, and *Igfbp1* genes, which are related to glucose metabolism, were increased in *Txndc5*-KO livers. Using an engineered hepatic cell line lacking TXNDC5, glucose uptake increased upon insulin stimulation. The response to the β-adrenoceptor agonist isoproterenol also increased in these KO cells. Taken together, the absence of TXNDC5 results in increased sensitivity to hepatic insulin action due to the insulin receptor’s preferential location on the plasma membrane.

The lower glycemia observed in males after 16 h of fasting suggests that TXNDC5 affects glucose metabolism in a sex-dependent manner. Interestingly, this effect occurred without changes in plasma insulin concentrations or HOMA, but coincided with higher plasma NEFA levels. In contrast, this phenotype did not occur over shorter fasting periods (Supplementary Table S2), and required prolonged fasting. These results contrast with previous studies on pancreatic β-cells in the absence of TXNDC5 [12,16], and evidence that harsher conditions are required to fully understand the effects of this protein. The observed decrease in glycemia may stimulate an increase in fatty acid release by peripheral tissues. Alternatively, the increased lipolysis could contribute to hepatic insulin resistance, compensatory hyperinsulinemia, or altered substrate partitioning, which modifies glucose. However, our data only allow us to rule out hyperinsulinemia.

The decreased glycemia observed following the OGTT is indicative of increased glucose uptake or decreased renal glucose reabsorption. Harsher conditions promoting a prediabetic state were explored using an HFHSD. On the HFHSD, male *Txndc5*-deficient mice also showed an improved OGTT response. Females showed no changes, consistent with the pattern observed under the chow diet. Taken together, these results suggest that insulin induces a stronger or faster signal in the absence of TXNDC5, and that this effect is weaker in females. Few studies have addressed the impact of sex on TXNDC5 [13]. A sexual dimorphic response regarding accumulation of hepatic squalene was observed in *Txndc5*-deficient mice [31]. Using pancreatic β-cell, estrogens were found to protect ER stress [32]. All of these previous findings combined indicate an interaction between TXNDC5 and sex that should be addressed. Contrary to previous articles that attributed a role in insulin folding to TXNDC5 [15], higher insulin concentrations were found in male KO mice consuming the high-fat, high-sucrose diet. The finding that lacking TXNDC5 provides glucose tolerance indicates a novel role for this protein.

This increased glucose tolerance led to the quantification of hepatic insulin receptor (INSR) levels, which showed no change in total homogenates but a surprisingly significant increase in the plasma membranes of TXNDC5-KO mice. Unlike in other tissues, hepatic INSR is believed not to be internalized following phosphorylation [33]. However, it shows a spatial preference in activating its downstream signaling by initiating the PI3K/AKT pathways on the plasma membrane [34]. This action terminates when the INSR is internalized via endocytosis, where it recruits targets, including IRSs, and activates the Ras/Raf/MEK/ERK pathway [34]. A higher INSR level on the plasma membrane due to impaired vesicle trafficking, resulting in a prolonged insulin response that favors the lower glycemia observed in the OGTT, is an attractive hypothesis to be proved. Consistent with this, an impairment in the management of lipid droplets containing squalene was observed in the absence of TXNDC5 [31]. Alternative explanations including reduced receptor internalization, altered degradation, altered recycling kinetics and changes in membrane stability are also possible and require further experiments. These will require female characterization because sexual dimorphism on the molecular mechanisms of insulin actions on adipose tissue has been described [35].

While *Insr* mRNA levels did not change, higher expression of *Irs2*, *G6pc*, and *Igfbp1* was observed in male *Txndc5*-deficient livers. IRS2 (insulin receptor substrate 2) is, along with IRS1, a substrate that transduces the INSR signal and activates the PI3K pathway [36]. IRS2 signaling dominates IRS1 signaling during the period directly after food intake and during prolonged fasting. It is more closely associated with hepatic lipid metabolism during fasting; the deletion of hepatic Irs2 results in mild fasting hyperglycemia [37,38,39]. The increased *Irs2* expression led to the slight hypoglycemia observed after 16 h of fasting. Glucose-6-phosphatase (*G6pc*) is an integral membrane protein of the endoplasmic reticulum (ER) that catalyzes the final step of gluconeogenesis and glycogenolysis by releasing glucose from the liver into the bloodstream [40]. The lower glycemia in *Txndc5*-deficient mice contradicts the hepatic increased *G6pc* expression. It should be noted that protein and enzyme activities were not assayed and posttranscriptional and posttranslational regulations exist. In addition, as we did not observe changes in glycemia during prolonged fasting, the late expression of gluconeogenesis genes might explain how glycemia was restored [41]. In addition to this role in blood glucose homeostasis, G6PC acts as a sensor of glucose and glucose-6-phosphate in the endoplasmic reticulum [42]. In this way, other glucose pathways could be influenced by the absence of TXNDC5. Finally, insulin-like growth factor-binding protein (IGFBP) regulates insulin-like growth factor (IGF) signaling by binding to the insulin-like growth factor 1 receptor (IGF1R) with an affinity equal to or greater than that of IGFs. Similarly, IGFs have structural similarities to insulin and can interact with INSR. An important function of circulating IGFBPs is to prevent this potential interaction because high concentrations of insulin-like growth factors in serum can cause hypoglycemic effects. Interestingly, IGFBP-1 was also found to increase in synovial fibroblasts when TXNDC5 was absent [5]. Hepatic IGFBP-1 expression is highly induced by starvation, hypoxia, stress, and proinflammatory cytokines, such as IL-1β, but is reduced by insulin [43]. IGF-1 reduces the release of non-esterified fatty acids (NEFAs) in adipocytes by inhibiting the action of growth hormone. It also reduces NEFA oxidation in the liver [44]. Higher levels of IGFBP-1 may sequester IGF-1, explaining the high plasma NEFA concentration observed in TXNDC5-KO mice. Changes in the expression of the *Irs2*, *G6pc*, and *Igfbp1* genes in the liver were significantly and negatively correlated with glycemia (Supplementary Figure S5), pointing to a possible connection, as previously discussed, that requires further experimental work.

The incubation of insulin on glucose uptake revealed that the TXNDC5-KO cell line took up significantly more glucose, which reproduced the previous in vivo findings. Isoproterenol, a non-selective β-adrenergic receptor agonist, induced a stronger lipolysis signal in the absence of TXNDC5. The action of isoproterenol is similar to that of glucagon [45]. If this in vitro finding occurs in vivo, then enhanced signal transduction could activate early glycogenolysis, explaining the lower glycemia and increased *Irs2* and *G6pc* expressions in the absence of TXNDC5.

Important limitations of this work include small sample size, absence of direct signaling pathway measurements (AKT, PI3K phosphorylation), no direct receptor trafficking assays and no hyperinsulinemic-euglycemic clamp studies.

## 4. Materials and Methods

### 4.1. Mice and Diets

Adult female and male wild-type and *Txndc5*-deficient mice on C57BL/6J genetic background were used and housed in sterile filter-top cages on a 12 h light/12 h dark cycle at the Centro de Investigación Biomédica de Aragón. All had ad libitum access to food and water. Mouse experiments were carried out in accordance with the EU Directive 2010/63 on the protection of animals used for scientific purposes, and the study protocol was approved by the Ethics Committee for Animal Research of the University of Zaragoza (PI35/18 and PI03/21).

Experimental diets used were a purified chow diet based on the AIN-93 diet for laboratory mice previously described [46]. This dietary intervention lasted four weeks. The purified high-fat, high-sucrose diet (HFHSD) was designed according to Burchfield et al. [21] and fed for five weeks. The latter diet was administered with a sucrose solution as the beverage and replaced weekly. All diets prepared in our facilities were lyophilized, and stored at −20 °C until use. Food intake and body weights were monitored every two weeks. At the end of the dietary interventions, mice were euthanized in a CO_2_ chamber, and tissue samples were collected and processed as previously described [14].

### 4.2. Functional Assays

For the oral glucose tolerance test (OGTT) and pyruvate tolerance test (PTT), mice were fasted for 16 h prior to the experiment. Oral glucose and pyruvate solutions (100 g/L) were administered at 2 g/kg body weight. Blood was collected from the submandibular vein at 0, 30, 60, and 120 min and processed. Animals were fasted for 6 h prior to the insulin tolerance test (ITT); intraperitoneal insulin (0.75 Units/kg body weight; Sigma, #91077C, St Louis, MO, USA) was administered. Blood was drawn from the submandibular vein at 0, 15, 30 and 60 min and processed as above. In long fasting tolerance tests, mice were fasted, and blood was collected from the submandibular vein at 0, 24, 36, and 48 h. At the last time point, mice were euthanized, and samples collected.

The use of different fasting durations (6 h for ITT, 16 h for OGTT/PTT and 48 h long fasting) was motivated by challenging stimuli, according to the current literature [47].

### 4.3. Plasma Assays and Analysis of Hepatic Lipids

Plasma glucose, total cholesterol and triglyceride concentrations were measured using Infinity^TM^ commercial kits (Thermo Scientific, Madrid, Spain). Plasma ketone bodies and non-esterified fatty acids were measured using kits (Fujifilm Wako Chemicals, Richmond, VA, USA). Plasma insulin was determined using an ELISA kit (RAI007R; RayBiotech, Peachtree Corners, GA, USA). Hepatic lipids were extracted using chloroform-methanol, dried, solubilized in iso-propanol and measured using Infinity^TM^ kit (Thermo Scientific).

### 4.4. RNA Extraction, cDNA Synthesis and mRNA Quantification

RNA extraction, cDNA synthesis, primer design (Supplementary Table S1) and qPCR were carried out as previously described [46].

### 4.5. Western Blotting Analyses

Preparation of hepatic protein extracts, quantification, and transference to a poly(vinylidene fluoride) membrane were performed as previously described [48]. The following rabbit polyclonal antibodies were used: anti-TXNDC5 (1:1000; Proteintech #19834-1-AP, Manchester, UK) and anti-INSR (1:500; FineTest# FNab04336, Wuhan, China). Equal loadings were confirmed by using a mouse monoclonal anti- β-ACTIN (1:2000; Sigma #A5441, St Louis, MO, USA). Membranes were washed three times with PBS buffer containing 0.1% Tween 20 and incubated for 1 h at room temperature with secondary antibodies goat anti-rabbit IgG (H&L) DyLight 800 (SA5-35575) and goat anti-mouse IgG (H&L) DyLight 680 (SA5-35518), both from Thermo Scientific, diluted 1/80,000. Images were captured using an Odyssey^®^Clx (LI-COR, Bad Homburg, Germany).

### 4.6. AML12 Cell Culture Assays

The wild-type and the Txndc5-deficient cell lines were previously described [23]. Seeding 15,000 cells/well in a 24-well plate was done to monitor cellular growth using cellular a Countess™ 3 FL Automated Cell Counter (Thermo Scientific). Cell viability assays were performed using an MTT viability assay as described [23]. Lipolysis and glucose uptake assays were performed as published [49].

### 4.7. Statistical Analysis

The G*Power 3.1.9.7 algorithm was used to calculate the sample size based on plasma triglycerides [50]. However, the calculated number exceeded the number of mice that the Ethics Committee would approve. Therefore, the sample size was adjusted to use the smallest possible number of animals while ensuring sufficient statistical power. This was accomplished by introducing group-homogenizing parameters such as breed, age, sex, body weight, and baseline plasma cholesterol.

Results are expressed as means ± SD. When the variables did not show normal distribution (according to the Shapiro–Wilk test) or failed to show homology of variance, comparisons were made using the Mann–Whitney U test using Prism 8 software for Windows (GraphPad, San Diego, CA, USA). This software was used to perform two-way repeated measures ANOVA on OGTT and ITT experiments. Associations among variables were sought using Spearman’s correlation tests. All calculations were performed using the Statistical Package for Social Sciences version 29 (IBM, Armonk, NY, USA). Significance was set at *p* ≤ 0.05.

## 5. Conclusions

These results indicate that TXNDC5 deficiency was associated with increased glucose tolerance and altered hepatic insulin receptor membrane localization. Contrary to other reports, the absence of TXNDC5 helped maintain glycemia at the prediabetic stage, accompanied by a surprising increase in plasma insulin levels. Furthermore, increased *Irs2* helps modulate insulin signaling. Finally, an elevation of plasma NEFA was found in KO mice, which was significantly correlated with glycemia. The increased *Igfbp1* may play a role in this correlation. Our results highlight the strong insulin response, in which INSR location is key to understanding the molecular mechanisms involved. However, more experiments are needed to better understand the new emerging hypotheses.

## Figures and Tables

**Table 1:** Somatometric and liver parameters in males.

	Wild-Type (n = 13)	Knock-Out (n = 10)
Mice weight (g)	28.8 ± 1.6	28.9 ± 1.2
Weight gained per food intake (mg/kcal)	15.6 ± 8.3	7.9 ± 5.9 **
Weight loss by 16 h fasting (%)	5.8 ± 0.9	5.5 ± 1.2
Liver mass (%)	3.9 ± 0.2	4.2 ± 0.3 **
Hepatic triglycerides (mg TG/g liver)	16.5 ± 1.7	19 ± 1.3 **
Hepatic cholesterol (mg cholesterol/g liver)	3.5 ± 0.6	4 ± 0.5 *

Mice were fed on a chow diet for 4 weeks and fasted 16 h prior to sacrifice. Data are means ± SD for each group. Statistical analyses were done according to Mann–Whitney’s U-test and *, *p* value < 0.05; **, *p* value < 0.01.

## Data Availability

All data will be made available on reasonable request.

## Supplementary Materials

The following supporting information can be downloaded at https://www.mdpi.com/article/10.3390/ijms27146286/s1.

## Abbreviations

The following abbreviations are used in this manuscript:

AUC	area under the curve
ELISA	enzyme-linked immunosorbent assay
HFHSD	high-fat, high-sucrose diet
HOMA	homeostatic model assessment
IGFBP	insulin like growth factor-binding protein
INSR	insulin receptor
ITT	insulin tolerance test
KO	knock-out
NEFA	non-esterified fatty acid
OGTT	oral glucose tolerance test
PDI	protein disulfide isomerase
PTT	pyruvate tolerance test
TXNDC5	thioredoxin domain containing 5
WT	wild-type
