# Supplementary material for: Absence of Thioredoxin Domain Containing 5 Improves Glucose Tolerance and Insulin Sensitivity in Male Mice
Source: Int J Mol Sci. 2026 Jul 15;27(14):6286. doi: 10.3390/ijms27146286 (PMC13410217; doi:10.3390/ijms27146286)
Supplement: Supplementary file 1 [file ijms-27-06286-s001.zip › ijms-4400951-supplementary.pdf]

Supplemental Figures

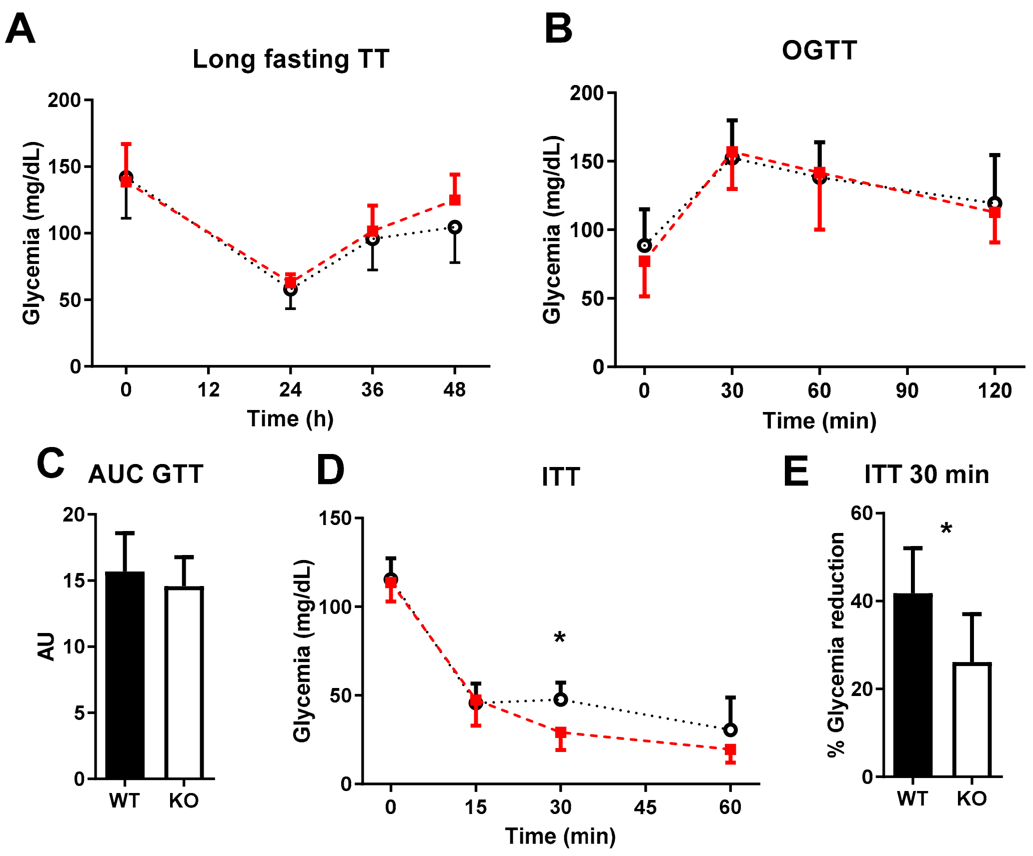

Supplemental Figure S1. Tolerance tests on female *Txndc5*-deficient mice. Glucose follow-up along prolonged fasting (A), oral glucose (B) and insulin (D) tolerance tests in adult *Txndc5*-deficient mice (KO) and wild type (n=6) used as a control group. Area under the curve of GTT (C) was calculated with no curve fitting taking baseline at 0 min. Reduction of glycemia at 30 minutes was plotted using ITT data (E). Data are means ± SD for each group. Statistical analyses were done according to Mann–Whitney U-test and *, p value < 0.05 vs WT.

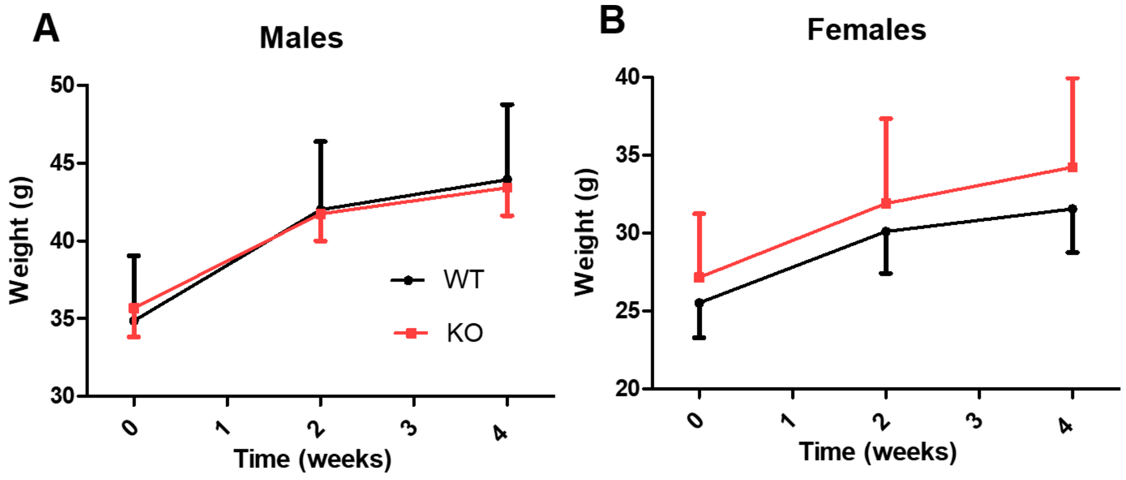

Supplemental Figure S2. Body weight follow-up of mice consuming a high-fat, high-sucrose diet during 4 weeks. Male (A) and female (B) adult *Txndc5*-deficient (KO) and wild-type mice (n=6). Data are means ± SD for each group. Statistical analyses were done according to Mann–Whitney U-test.

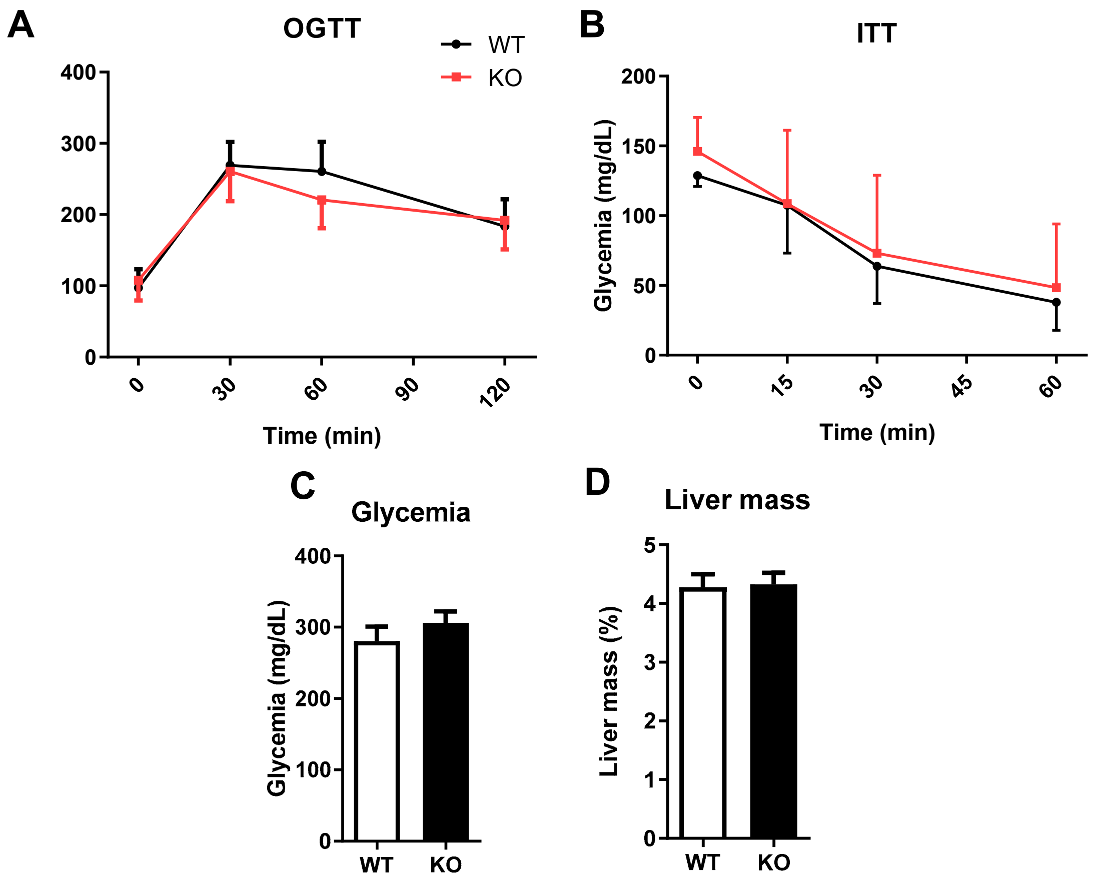

Supplemental Figure S3. Tolerance tests, plasma, and hepatic parameters of female mice on a high-fat, high-sucrose diet (HFHSD). Oral glucose tolerance test (A), insulin tolerance test (B). Liver mass (C) and plasma glucose (D) were measured after 16-hour fasting. Data are means ± SD for each group (n= 6 and n = 7, respectively for WT and *Txndc5*-deficient, KO). Statistical analyses were done according to Mann–Whitney U-test.

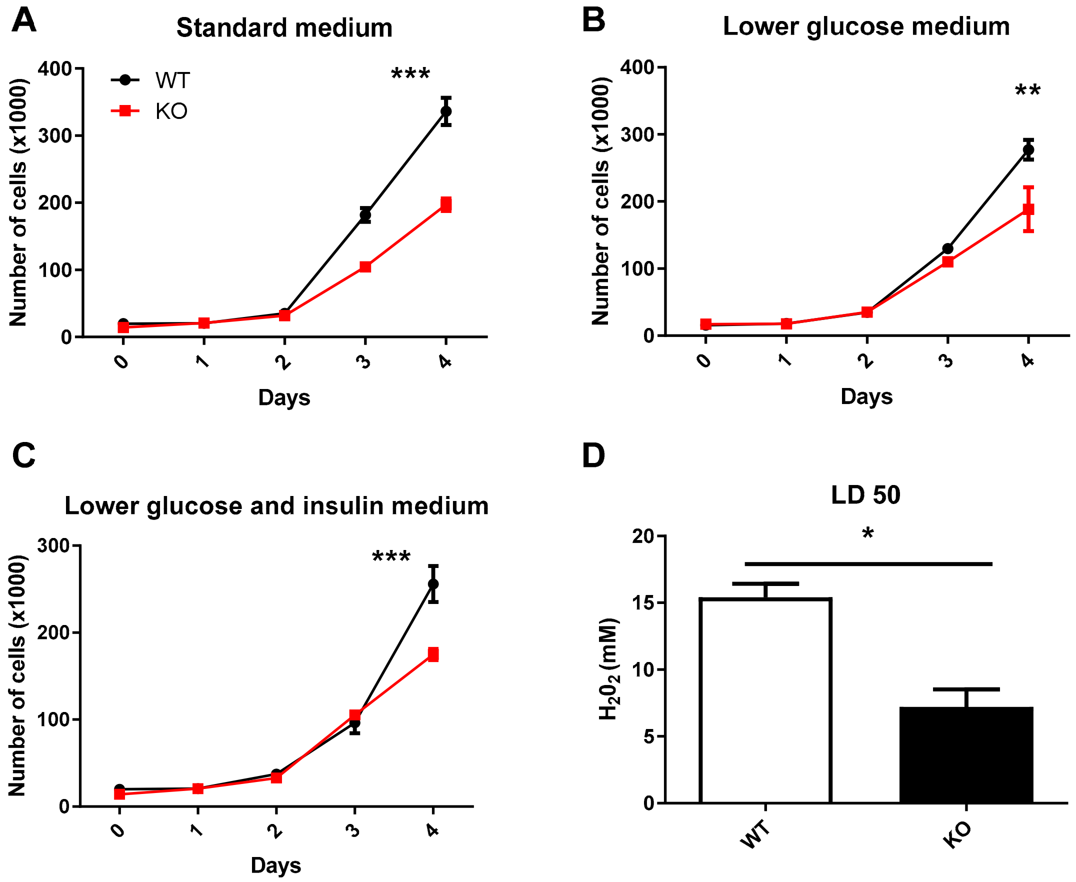

Supplemental Figure S4. Characterization of *Txndc5*-deficient AML12 cell line. (A-C) Growth curve of AML12 under several cell culture media. (D) Lethal dose 50 under a treatment of 30 minutes with hydrogen peroxide. Data are means ± SD for each group (n= 4). Statistical analyses were done by Kruskal-Wallis ANOVA and Mann-Whitney U-test as post-hoc comparisons. *, $P<0.05$, **, $P<0.01$ and ***, $P<0.001$

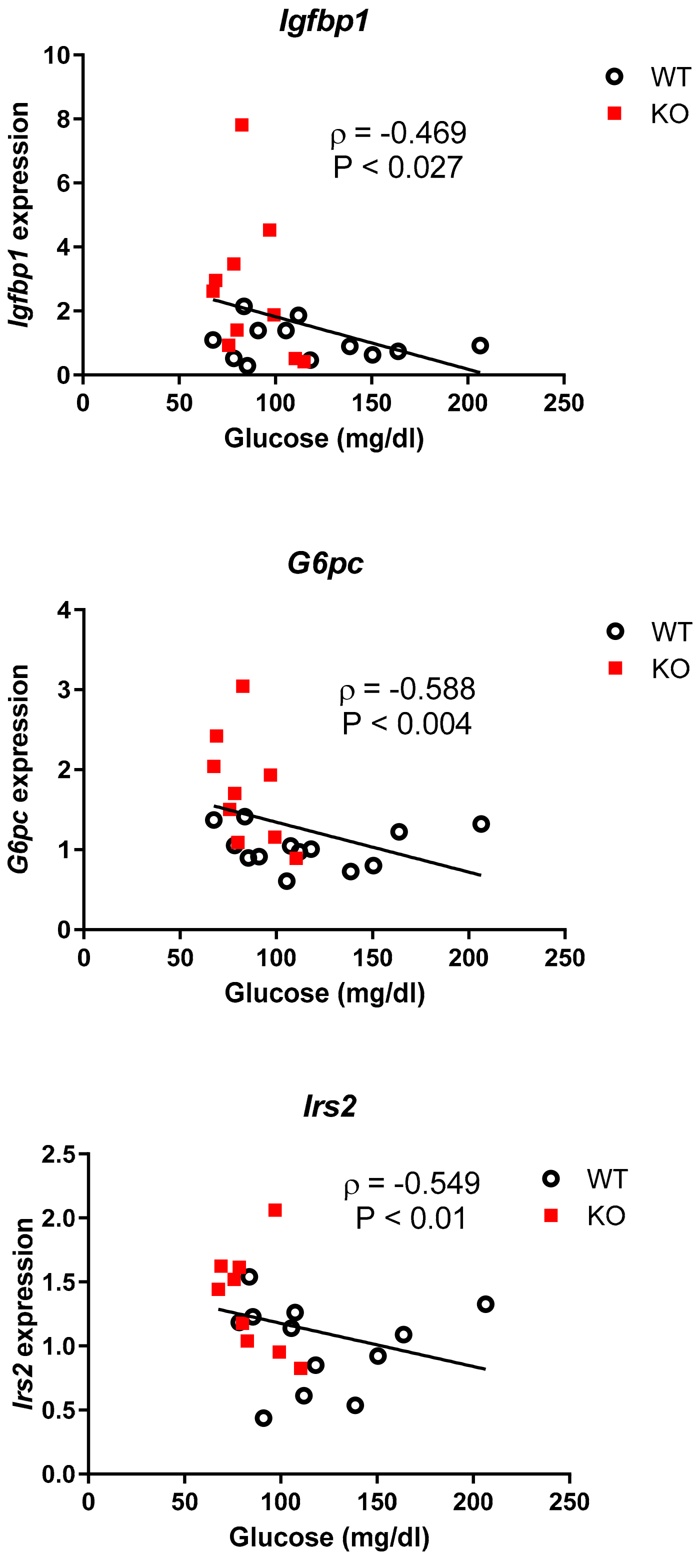

Supplemental Figure S5. Correlation among hepatic gene expressions and glucose concentrations in males. Spearman correlation coefficients and their significance are displayed.

Supplemental Tables:

Supplementary Table S1. Characteristics of murine primers used in RT-qPCR assays following MIQE guidelines

Gene symbol	Accession	Sequence		Amplicon length	Junction	[Primer]	Efficiency
G6pc1	NM_008061.4	Forward:	AGACTCCCAGGACTGGTTCA	199	Exon 1/2	100 nM	109%
		Reverse:	GTCCAGGACCCACCAATACG				
Igfbp1	NM_008341.4	Forward:	TCGCCGACCTCAAGAAATGG	206	Exon 5/6	100 nM	106%
		Reverse:	CCATGGGTAGACACACCAGC				
Insr	NM_001330056.1	Forward:	CTGTGTCTCCCGGAAGCATT	173	Exon 13/14	200 nM	102%
		Reverse:	TGGCAATATTTGATGGGACATCT				
Irs2	NM_001081212.2	Forward:	TGAAGGAAGCCACAGTCGTG	142	Exon 2/3	200 nM	96%
		Reverse:	GTTGGTCGGAAACATGCCAA				
Ppib	NM_011149	Forward:	GGAGATGGCACAGGAGGAA	72	Exon 3/4	100 nM	98%
		Reverse:	GTAGTGCTTCAGCTTGAAGTTCTCAT				
Txndc5	NM_145367.4	Forward:	CAGGCTTGTCAGATGTCACCAT	82	Exon 8/9	200 nM	100%
		Reverse:	TAACCTCGTACCGAGTACTTGCTG				

Supplemental Table S2. Plasma parameters at 6-hour fasting.

	Males		Females		Two-way ANOVA		
	Wildtype (n= 13)	Knock-out (n= 10)	Wildtype (n= 5)	Knock-out (n= 14)	Genotype	Sex	Interaction
TG (mM)	1 ± 0.2	1 ± 0.1	0.8 ± 0.2	0.8 ± 0.2	NS	P=0.002	NS
Cholesterol (mM)	2.7 ± 0.4	2.9 ± 0.3	1.9 ± 0.2	1.9 ± 0.3	NS	P<0.0001	NS
Glucose (mg/dL)	194 ± 33	196 ± 32	151 ± 13	153 ± 38	NS	P=0.0004	NS

Mice were 4 weeks on a chow diet and fasted for 6 hours before sampling. Data are means ± SD for each group.

Statistical analyses were done according to Mann–Whitney U-test to compare wildtype vs knock-out. Two-way ANOVA was used to assess genotype, sex, and their interaction.

Supplemental Table S3. Plasma parameters at 16-hour fasting.

	Males		Females		Two-way ANOVA		
	Wild-type (n= 13)	Knock-out (n= 10)	Wild-type (n= 5)	Knock-out (n= 14)	Genotype	Sex	Interaction
TG (mM)	1.8 ± 0.3	2 ± 0.5	1.1 ± 0.3	1.2 ± 0.2	NS	P=0.0001	NS
Cholesterol (mM)	2.7 ± 0.4	3 ± 0.2	2.3 ± 0.5	2.1 ± 0.4	NS	P=0.0001	NS
Glucose (mg/dL)	116 ± 39	87 ± 17*	129 ± 38	130 ± 41	NS	P=0.02	NS
NEFA (mM)	1.9 ± 39	2.5 ± 17***	1.6 ± 0.2	1.6 ± 0.2	P=0.0016	P<0.0001	P=0.0016

Mice were 4 weeks on a chow diet and fasted 16 hours prior sampling. Data are means ± SD for each group.

Statistical analyses were done according to Mann–Whitney U-test to compare wildtype vs knock-out. *, P<0.05 and ***, P < 0.0001 vs WT. Two-way ANOVA was used to assess genotype, sex, and their interaction.

Supplemental Table S4. Plasma parameters at 16-hour fasting under HFHSD for 4 weeks.

	Males		Females		Two-way ANOVA		
	Wildtype (n= 6)	Knock-out (n= 6)	Wildtype (n= 6)	Knock-out (n= 6)	Genotype	Sex	Interaction
TG (mM)	0.6 ± 0.1	0.6 ± 0.2	0.4 ± 0.1	0.4 ± 0.1	NS	P=0.0014	NS
Cholesterol (mM)	5 ± 0.3	4.9 ± 1	2.8 ± 0.5	3.2 ± 0.5	NS	P=0.0001	NS

Mice were 4 weeks on a HFHSD diet and fasted for 16 hours before sampling. Data are means ± SD for each group. Statistical analyses were done according to Mann–Whitney U-test to compare wildtype vs knock-out.
